# The politics of agenda setting at the global level: key informant interviews regarding the International Labour Organization Decent Work Agenda

**DOI:** 10.1186/1744-8603-10-56

**Published:** 2014-07-01

**Authors:** Erica Di Ruggiero, Joanna E Cohen, Donald C Cole

**Affiliations:** 1 Dalla Lana School of Public Health, University of Toronto, Health Sciences Building, 155 College St., Toronto, Ontario M5T3M7, Canada; 2 Johns Hopkins Bloomberg School of Public Health, 2213 McElderry, 4th Floor, Baltimore, Maryland 21205, USA; 3 Global Health Division, Dalla Lana School of Public Health, University of Toronto, Health Sciences Building, 155 College St., Toronto, Ontario M5T3M7, Canada

**Keywords:** Policy, Agenda setting, Work, International agencies, Qualitative research

## Abstract

**Background:**

Global labour markets continue to undergo significant transformations resulting from socio-political instability combined with rises in structural inequality, employment insecurity, and poor working conditions. Confronted by these challenges, global institutions are providing policy guidance to protect and promote the health and well-being of workers. This article provides an account of how the International Labour Organization’s Decent Work Agenda contributes to the work policy agendas of the World Health Organization and the World Bank.

**Methods:**

This qualitative study involved semi-structured interviews with representatives from three global institutions – the International Labour Organization (ILO), the World Health Organization and the World Bank. Of the 25 key informants invited to participate, 16 took part in the study. Analysis for key themes was followed by interpretation using selected agenda setting theories.

**Results:**

Interviews indicated that through the Decent Work Agenda, the International Labour Organization is shaping the global policy narrative about work among UN agencies, and that the pursuit of decent work and the Agenda were perceived as important goals with the potential to promote just policies. The Agenda was closely linked to the World Health Organization’s conception of health as a human right. However, decent work was consistently identified by World Bank informants as ILO terminology in contrast to terms such as job creation and job access. The limited evidence base and its conceptual nature were offered as partial explanations for why the Agenda has yet to fully influence other global institutions. Catalytic events such as the economic crisis were identified as creating the enabling conditions to influence global work policy agendas.

**Conclusions:**

Our evidence aids our understanding of how an issue like decent work enters and stays on the policy agendas of global institutions, using the Decent Work Agenda as an illustrative example. Catalytic events and policy precedents were found to contribute positively to agenda setting. Questions remain, however, across key informants about the robustness of the underlying evidence base for this Agenda and what meaningful impacts have been realized on the ground as a result.

## Introduction

Global labour markets continue to undergo significant changes as part of globalization. These transformations range from greater access to technological advancements that hold the potential to improve the quality of people’s lives to increases in employment opportunities. Recent decades are also marked by transformations resulting from socio-political instability combined with rises in structural inequality, employment insecurity, and poor working conditions [1]. In response to these profound changes, some global institutions use policy instruments to encourage multiple actors to improve decent working conditions. In the context of globalization, global institutions operate in an increasingly complex and contested space. Given the recent economic crisis, scholars are calling for greater attention to the effective functioning of global institutions [2]. The capacity, power and actions of these actors – and their interactions – offer a “site” for understanding the evolution and regulation of work globally [1]. Among global institutions one can study horizontal policy agenda setting processes – how they mutually influence each other to set agendas and what value they contribute to the policy process. One such global institution, the International Labour Organization (ILO), has a mandate to promote social justice and internationally recognized human rights, specifically those in relation to labour. Established in 1919, the ILO was originally envisioned as an “instrument of international social reform” and a “scientific and impartial body” independent of states [3]. The ILO includes tripartite representation from employers, workers and state representatives, which according to the ILO provides it with “an edge in incorporating real world knowledge about employment and work” into the policy development process [4].

The purpose of this article is to provide a qualitative account of how the ILO Decent Work Agenda^a^ contributes to the work policy agendas of two other institutions – the World Health Organization (WHO), which like the ILO operates within the UN system, and the World Bank (WB), which is not formally part of the UN system. It discusses the results from key informant interviews conducted with the ILO, WHO and World Bank representatives, which shed light on the processes that contribute or hinder policy agenda setting. The results point to the need for institutions such as the ILO to be nimble and adaptive to events and to a shifting global, political and economic context, in order to influence policy agendas that promote workers’ well-being.

We start with a brief description of the evolution of the Decent Work Agenda and theoretical approaches to agenda setting. After describing the methods, we set out our findings under key themes emerging from the interviews. We discuss these in light of selected theoretical approaches to agenda setting, and share implications of our findings and potential future directions.

### Background

#### ILO Decent Work Agenda in an era of globalization

In 1999, the ILO launched the Decent Work Agenda (DWA or simply the Agenda) to guide the development of policy that aims to protect and promote workers’ well-being around the globe. The Agenda encourages all nations to offer women and men the opportunity to work in freedom, equity, security and human dignity. ILO defines decent work as the sum of the aspirations of people for “opportunity and income; rights, voice and recognition; family stability and personal development; and, fairness and gender equality” (ILO definition). Through the Agenda, the ILO wants to ensure that workers have equal access to work that is safe, secure, sustainable and productive, respects a person's fundamental rights at work and provides the freedom to voice one’s concerns. The ILO asserts that these objectives can only be achieved through “decent work-oriented approaches to economic and social policy in partnership with the principal institutions and actors of the multilateral system and the global economy^a^.”

Decent work matters in terms of health. Work that is not decent can produce significant adverse health effects through material and social deprivation and unsafe working conditions [5]. Work-related accidents or diseases account for more than 2.3 million deaths per year, and 317 million accidents, which in turn result in many absences. The related human and economic costs are estimated at 4% of the global Gross Domestic Product each year [6]. Studies are also showing that work intensification and non-standard employment (e.g. informal work) are linked to poor health and social outcomes [1,5]. Decent work is also not equally and equitably accessible to all individuals, communities or nations. The effects of globalization provide a partial explanation. The current form of globalization has been characterized as “not a natural or inevitable fact but a series of deliberate decisions that disproportionately favour some over others” [7]. Globalization transcends national boundaries to affect health in several ways. A growing body of evidence suggests that it is “giving rise to new patterns of health and disease linked to the consequent restructuring of human societies” [8]. It is transforming socioeconomic, cultural and environmental conditions and contributing to the reconfiguration of existing health challenges, including health inequalities within and between countries [8]. In the face of the most recent economic crisis, the now former ILO’s Director General, Juan Somavia denounced the “structural imbalances of the current model of globalization….a model that during the last three decades has overvalued the role of the market, devalued the role of government, and diminished the dignity of work and respect for the environment” [9].

The ILO has tried to promote decent work through the policy process. Since the launch of the Decent Work Agenda in 1999, the ILO has, for instance, raised awareness of the importance of decent work through the UN, G20 and other global fora, disseminated policy documents (e.g. Social Protection Floor Initiative), and provided technical guidance on topics such as promising employment and social policies that promote decent work conditions [10]. That said, these efforts have not been without their challenges. Heymann and Earle [11] assert that “not everyone believes that we have a strong obligation to ensure a minimum floor of working conditions and equal opportunity for all human beings.” We must interrogate the discursive root causes of such policy perspectives. In particular, how can a policy instrument such as the Decent Work Agenda contribute to both workers’ well-being and to fair globalization by, as the ILO has framed it, “harnessing [its] benefits while promoting sustainable economic and social development” [12]?

#### Agenda setting

The study of agenda setting processes deepens our understanding of how an issue such as decent work lands on the policy agendas of global institutions in the first place. The study of agenda setting involves asking how health and social problems emerge and stay on the policy agenda, how policy makers become aware of these problems, how attention and resources are allocated to these problems, and how agendas are set and produced through political interactions of social actors [13-15]. Language and practices shape, legitimize and support policy agendas in discourses and interactions between organizations.

Kingdon argues that “agenda setting has a random character in which problems, policies and politics flow along [three] independent streams” [14]. He further suggests that only once there is convergence across the three streams (problems, policies, and politics) can a problem or issue emerge on the policy agenda. Other scholars, such as Baumgartner and Jones, have argued that agenda setting implies “no one” single equilibrium in politics [16]. Rather it is characterized by stability and rapid transformation. In periods of stability, agenda setting is a more integral part of the policy process, more incremental in nature and more likely influenced by precedents (e.g. past policy solutions, long-standing collaborative agreements between organizations).

Agenda setting can also be marked by periods of volatile change [16]. Catalytic events (e.g. economic crisis) provoke disruptions in the policy equilibrium and can lead policy makers and politicians to pay greater attention to the problems highlighted by these events and can trigger convergence between problems, policies and politics [15,16]. Agenda access also comes into play in periods of stability and change [16]. Taken together, these insights from the literature point to different processes that might explain how policy agendas are established, in the first place, and then mobilized through the actions of institutions. Although the focus has shifted away from linear and incremental descriptions towards approaches that recognize the iterative and dynamic nature of agenda setting processes, less is known about how health and social issues get and stay on the policy agendas of global institutions, and how organizations influence each other to set global policy [15]. Global institutions and the interactions between them provide an important site of study for understanding the latter horizontal policy agenda setting processes. Our study attempts to shed light on the processes at work in policy agenda setting using the Decent Work Agenda as an illustrative example.

## Methods

This article reports the results from sixteen semi-structured interviews with representatives from three global institutions – the ILO, WHO and World Bank. These organizations were chosen because of their major policy leadership around work, health and/or economic development, respectively. The interviews aimed to identify how these three organizations contribute to policy agenda setting and to document the forms of knowledge, social norms, rules and claims (e.g. economic, social, health), which affect different organizations’ conceptualizations of decent work.

### Recruitment

A purposive sampling approach was used to recruit 5–6 representatives per organization to examine a diversity of perspectives within and across the ILO, WHO and World Bank. These key informants (KIs) were chosen on the basis of their ability to provide relevant insights about the topic under study [17]. The following selection criteria were used to select KIs: 1) individuals with exposure to the topic and content expertise about decent work and/or labour policy; 2) individuals in the WHO and World Bank with an established relationship to the ILO, including those who collaborate or operate at the interface of their organization and the ILO, or have some knowledge of this relationship (e.g. individuals who have worked on a joint policy document with the ILO); and, 3) individuals who work at different policy and program levels within each organization. Prior to recruitment, ethics approval was sought from and granted by the Office of Research Ethics at the University of Toronto (Health Sciences Research Ethics Board).

A total of twenty-five individuals were invited to participate by e-mail between October 2011 and May 2012 using a standard recruitment letter, which included information about the study and the consent form. Some participants were already known to the lead author based on preliminary consultations with two of the three organizations at the study design stage. Others were identified through a combination of referral and snowballing techniques following the conduct of initial interviews [18,19].

Of the twenty-five contacted, sixteen people agreed to take part in the study. Some challenges were encountered with participant recruitment. Some individuals did not respond despite several follow-up attempts by email and phone or they declined to participate either because they did not have the time due to competing commitments, overseas travel and/or organizational restructuring, or they did not feel they were the ‘right person’ (i.e. had insufficient knowledge to contribute to study; did not feel their organization could comment on decent work issues). Those who declined were encouraged to recommend others to participate, and in some cases, they suggested someone else within their organization.

Eleven semi-structured interviews were conducted in person in Geneva (ILO, WHO) and Washington D.C. (World Bank), and the remaining five by phone between November 2011 and May 2012. Six from the ILO, five from the World Bank and five from the WHO took part. A total of 9 men and 7 women participated. Interview participants had worked in their current organization (but not necessarily in their current position) from two to 28 years. KIs represented different disciplinary backgrounds (e.g. occupational health, labour economics, social policy, gender and development), held positions at different levels (from junior positions to senior management) and performed different roles (e.g. technical specialists; partnerships, policy and research roles).

### Data collection

A semi-structured approach was used to explore common issues while allowing for topics of relevance to emerge from key informants’ interests or experiences during the interviews. This approach to interviewing provided each KI the chance to describe and frame issues from their perspective and permit the emergence of ideas that are outside of the researcher’s own theoretical framework [20]. Table 1 provides examples of core questions from the interview guide, which was adapted for use with each organization. Interviews ranged in length from 40 to 75 minutes and were all conducted in English. The interviews covered topics such as individual and organizational perspectives on decent work, global influences on decent work policy, perceived meanings of the Decent Work Agenda, involvement and ownership in the Agenda, as well as its current and future relevance. The same investigator (lead author) conducted all interviews to enhance consistency. All participants were encouraged to recommend others within or from another organization that was part of the study. Data collection was completed following exhaustion of all recruitment options, that is after no other participants could be identified through referrals or snowballing techniques [18,19].

**Table 1:** Sample Interview Questions

**Domain**	**Illustrative Probes**
Perspectives on decent work	How is decent work important to you/your organization?
	What does decent work mean to you/your organization?
	How was it decided that the term “decent work” should be used by your organization? When did this occur? Why do you think this happened?
Decent Work Agenda	Who owns the Decent Work Agenda? Has this changed since it was first in launched in 1999? How?
	Who else supports the Decent Work Agenda? Why?
	Who might oppose the Agenda? Why?
	Looking ahead, what role do you see the Decent Work Agenda playing in the next five years?

### Analysis

The interviews were transcribed verbatim, validated against the recording, and the data stored in a password protected location. Interview data were coded using ATLAS.ti 6.2 qualitative data management software. A preliminary hermeneutic unit and coding scheme were constructed, informed by the study research questions. Codes were generated through a combination of very descriptive (close to the data) codes and more conceptual ones informed by the literature [20]. Through an iterative process, codes were eliminated and a thematic analysis was conducted to identify overall patterns in the data. Then links were made to the study research questions and relevant literature on agenda setting and policy. By analyzing participant accounts thematically, similarities and differences could be contrasted in order to provide a better understanding of the nature of collaboration, policy agenda setting processes, and contributions of each of these to the setting of work policy agendas at a global level.

## Findings

The themes identified herein reflect enabling and unsupportive agenda setting influences that relate to the saliency of decent work and the perceived sense of ownership in the Agenda, the nature of collaboration, congruence and coherence of different organizational policy agendas, and the role of catalytic events. Together, they shed light on the role of the Decent Work Agenda (DWA) in setting the global policy agendas of the WHO and World Bank. They also unpack the nature of the interactions between these three organizations, and point to influences of the socio-political and organizational context in which the Decent Work Agenda is operating.

### Saliency of decent work

The interviews identified that through the DWA, the ILO is shaping the global policy narrative about work and therefore setting this global agenda. There was general agreement among key informants from all three organizations that the pursuit of decent work and the Decent Work Agenda were laudable goals with the potential to promote just policies. The extent of agreement was even higher when a KI commented from an individual perspective.

“*Any normal human being would like everybody to have access to a decent job.... I guess the differences are more in terms of what needs to be done.”* (WB KI)

The Agenda was identified as a “leitmotif” that guides policy activity for the ILO in particular, a political text, a slogan or a sort of shorthand that could succinctly communicate concepts about decent work (ILO and WHO KIs). These included acceptable, respectable, adequate, healthy, safe, and dignified work relative to one’s local circumstances that provides a sense of purpose.

“[Decent work] touches upon the core business of this organization, which is health. (WHO KI)

The decent work (DW) concept was also seen as more embracing of different work arrangements compared to the word ‘job’ (ILO KI). Inherent tensions such as decent work is supposed to combine economic efficiency with social justice were mentioned by some KIs.

In contrast, all World Bank KIs referred to decent work as “ILO’s terminology” and consistently reported not using it. Instead, they used terms such as good and quality jobs, better work, job creation, job access and equal opportunities for jobs^b^. Several WB KIs noted that because 80% of work is informal especially in developing countries, the definition of ‘decent’ in those conditions is very difficult to measure, let alone enforce. They consistently reported the Bank focusing on jobs as a means to reduce poverty:

*“You first need to have a paid job; then, you can worry about it being formal and then you can worry about it being decent” (WB KI)*.

Some WB informants did, however, remark that “good jobs may actually not be decent, according to the standard definition of decent employment from the ILO”. Some also noted that a few dimensions of the DW Agenda (social protection, employment) do map onto the Bank’s work related programs (e.g. social protection, better work program, gender and development initiatives).

WHO informants identified the DW Agenda with the ILO and explicitly noted several points of congruence with their objectives. The Agenda was seen to be closely aligned with the WHO’s conception of health as a human right. A WHO KI identified a natural fit of fair employment and decent work with social determinants of health, health equity and health in all policies frameworks, which the WHO uses. Finally, the Agenda did influence to some extent the organization’s thinking in relation to human resources. It was identified as a “very useful point of reference” (WHO KI) to guide policy. It was also seen as carrying particular weight coming from a specialized agency (i.e. ILO) within the UN system.

### Perceived sense of ownership

When asked who owns the Decent Work Agenda, all KIs pointed without hesitation to the ILO though in one instance the UN Chief Executive Board was also named. The ILO has carved out a distinctive niche through the Decent Work Agenda – it provides a reasonably coherent explanation of ILO objectives and a common platform for working with other international organizations. Organizational mandate was seen as inextricably linked to ownership as were the interests of key constituencies (e.g. member states, workers and employer organizations). Yet some ILO and WHO KIs reported that many agencies with whom they work can be extremely “territorial in trying to protect their niche”. This arises according to one informant because the DWA overlaps with ‘hot topics’ such as the social determinants of health, social protection, and the Millennium Development Goals where every agency is trying to put their spin on the global agenda and compete for political attention and resources. It can also manifest through an organization’s actions. In reference to the World Bank’s Jobs Development Report (note: only the report’s outline was publicly available at the time of these interviews), one ILO KI remarked:

“How come they wrote all that without saying decent work? … that’s sort of part of the, the problem that if you’re doing a flagship report at World Bank, you could have your own flag. But … the fact {is} that the intellectual content of the Decent Work Agenda is influencing the World Bank’s thinking.”(ILO KI)

### Nature of collaboration

All informants reported some level of collaboration with the other two organizations in question with the greatest and most consistently reported collaboration between the ILO and the WHO. Reasons given included the fact that the ILO and the WHO are both formally part of the UN system, the role of historical precedents such as constitutional agreements, and the existence of collaborative structures such as joint technical steering committees or inter-agency initiatives (e.g. Social Protection Floor Initiative [21]; Joint WHO/ILO/UNAIDS policy guidelines [22] for health care workers). Some KIs cited more recent examples of collaboration with the World Bank at the country level (e.g. sharing information about needs assessments and declared states of emergency where the WB is acting as trustee of funds). This may suggest that the context for collaboration, and the potential for incremental change and mutual influence of agendas, is high.

### Congruence between the ILO Decent Work Agenda and the organizational objectives of WHO and World Bank

Several KIs reported inherent tensions and trade-offs such as balancing social and market needs and the impacts of limited budgets on their organizations in trying to advance ***a*** common work agenda globally. Territorialism and turf battles characterize this dynamic and increasingly competitive and fiscally constrained environment where global institutions need to redefine their core business and functions in relation to each other. While for some this took the form of disengagement (i.e. “decent work” is the ILO’s business), others readily reported common ground with the concept of decent work and the DWA. For instance, a WHO key informant reported the use of “‘health of workers’ or ‘workers’ health’ or also ‘occupational health’ not decent *per se* although they pursue the same goals as the ILO obviously, but on a different scale”. Another WHO KI noted the following:

“The big advantage [of the Decent Work Agenda] is the integration aspect, the fact that it does link right across the whole area of work… [for example] social protection, which the ILO’s always been very strong in… seeing how we linked social protection into other policies”

Policy precedents affect whether an issue is considered a priority for an organization and how it is framed. For instance, some World Bank informants mentioned that the Bank did not always see labour as a big priority given interests in poverty reduction in developing countries. However, this is changing and opportunities for collaboration between the ILO and WB (e.g. social protection) are emerging.

“The ILO is more oriented to looking at the labour market and particularly the formal labour markets whereas the Bank’s emphasis is always, you know, the poor and this makes a bit of difference in the approach but, but I would say the relationship today is much more cooperative.” (WB KI)

There is evidence that the Decent Work Agenda also impacted agenda access, which occurs when an issue mobilizes greater numbers of constituencies [16]. Some KIs reported ignoring the Agenda or parts of it because they were not convinced that it was grounded in robust evidence. For example, from an organizational perspective, some World Bank informants noted having difficulty engaging with the workers' rights agenda (which is one of the four core strategic areas of the DWA). However, they went on to report that in cases where there is empirical economic evidence suggesting that decent work is good for development and long term growth rates, and has quantifiable economic impacts, then it can be supported. The example given was that of child labour, which had been empirically shown according to one informant to reduce human capital development.

“Within the Bank Group, very, very little focus on any of the rights aspects to jobs, for sure. It’s all viewed through a lens of what promotes development” (WB KI).

### Policy coherence

Several ILO and WHO informants highlighted how the DW Agenda contributed to greater policy coherence within the family of United Nations (UN) Agencies. WHO informants commended the ILO for continually raising the issue of decent work with the Chief Executive Board, where all heads of UN agencies come together. The ILO’s persistence in navigating global governance mechanisms such as the “UN system-wide Coherence Process” led to the successful adoption of the DW Agenda by more than 30 affiliated agencies^c^. KIs also noted several policy documents and declarations that reiterate the key messages of the DW Agenda. However, a frequently cited limitation of “decent work” and the Decent Work Agenda’s discursive role across all three organizations was that it has remained too conceptual, that it is difficult to measure, that it lacks a robust evidence base (in particular economic evidence), and that it has yet to result in a completely coherent, comprehensive policy strategy. In one ILO informant’s view, this policy rhetoric had absolutely no impact. According to one WB informant, this is a matter of political economy. At a national level, a complex set of multi-level policies need to be coordinated to create and improve access to good quality jobs yet there is very limited communication between the different ministries managing them. The policies include macro-economic policies that affect investments, labour regulations, passive and active labour market programs, education policies that affect the distribution and the supply of skills, and policies dealing with social protection. The lack of communication and coordination pose challenges for increasing the quality and quantity of jobs that are business friendly and open to and benefit the majority of workers.

According to both ILO and WB informants, more sensitive indicators were felt to be needed that extended beyond just employment and unemployment rates to include ‘softer’ indicators (e.g. the relationship between work and social cohesion was seen as less direct). The limited evidence base was offered as a partial explanation for why the DW Agenda has yet to really influence policy making at the country level. In contrast, some informants (WB, WHO) highlighted one of the many conundrums faced by global institutions in setting national and local policy agendas.

“We are supposed to set norms and standards…” “Our role is … to work through the regions and countries to implement those norms and standards and work with them on pilot demonstration projects … and so, sort of by definition, we are removed from the real impact of our work at the ground level”. (WHO KI)

### Catalytic events

KIs mentioned catalytic events, which can create windows of opportunity and enabling political, economic and social conditions for influencing policy agendas. The arrival of a new leader, Juan Somavia at the ILO back in 1998, led to the introduction of the DW Agenda and its approval by the ILO Governing Body and at the Conference. The Organization then adopted decent work as a modern version of its historical mandate. More recently, the global economic crisis and related consequences also had a catalytic effect. The ILO capitalized on this window of opportunity by working with the G20 to put decent work back on the agenda of these countries, as substantiated by these informant quotes:

“… In 2008, the labour conference adopted the ILO Global Jobs Pact which is a menu of options, of policies that have proved to work well in the situation of crisis response but also in … a normal situation. So the recent economic crisis, the Global Jobs Pact did sort of this for the Decent Work Agenda but translated [it] into sort of a crisis response package” (ILO KI)

“What’s happened with the first crisis of globalization and the recognition that in that boom period before the bust, employment wasn’t growing that well, even in the good times, and now in the bad times it’s serious trouble … I think there is recognition that, you know, we can’t just sort of cross our fingers and hope that other forces will create the conditions which enable decent jobs to be fostered. We’ve actually got to do it in a much more integrated way with employment. Much more near the heart of the economic thinking! And that’s been the biggest change in the last few years.” (ILO KI)

“I think the most recent work at the G20 level was really important to highlight the necessity to work on these aspects, but it was probably not enough because for the time being, the G20 are considering decent work as important probably because of the crisis context, but decent work is of course not only a crisis concept, … it is a concept that should apply to all situations …”(ILO KI)

## Discussion

Study results point to evidence of agenda setting regarding the Decent Work Agenda. These include evidence of: saliency of decent work as an issue worthy of pursuit; congruence between the Agenda and the organizational objectives of others (e.g. fit between decent work and social determinants of health and health as a human right); and, policy coherence through shaping of policy discourses and practices. For instance, KI data suggest that policy coherence has in part been enabled by organizational precedents, joint collaborative structures and endorsements by influential bodies such as the UN Chief Executive Board and the G20. These processes have contributed to putting decent work on the agendas of these global institutions and governance mechanisms.

Fundamental to an understanding of agenda setting is knowledge of vested interests and how these are shaped by organizational ideologies and values and can lead to resistance. A key theme arising is the World Bank’s resistance to the language of ‘decent work,’ at least in part because it is perceived to conflict with market imperatives. They also seem to resist any parts of a rights framework that they do not perceive to be based on evidence, further suggesting a more ideological component to their position. The World Bank’s decision to not adopt the language of the Decent Work Agenda could be interpreted as an attempt to redefine the discourse of work and exert their power and influence to occupy this policy space internationally. Regardless of what WB KIs might say, indeed, the Bank’s policies in labour market flexibility (including unrestricted international flows of labour) actually conflict with the promotion of decent work conditions. For example, the WB key informant’s assertion that countries must get their people to have paid work first, then informal employment and then formal employment is not consistent with the global evidence base. Benach and colleagues [5] documented the opposite in poor countries where the shift away from informal to formal employment did not occur; instead, there was often a growth in informal employment.

That said, the ILO’s long-standing focus on improving decent working conditions may have indirectly raised the profile of labour issues within the WB over time, and ultimately led to its 2013 World Development Report on Jobs [23]. While some progress has been made, questions remain according to KIs across all three organizations about the robustness of the underlying evidence base for the DW Agenda and also what impacts have been realized on the ground as a result. This limited impact is perhaps not surprising in the case of organizations such as the ILO who establish agendas that conflict with dominant thinking, including the neoliberal discourses of more powerful agencies such as the International Monetary Fund (IMF) and the World Trade Organization (WTO). Over the last fifteen years, the ILO Decent Work Agenda, labour standards and conventions have been incorporated into various UN and country-level policy documents, guidelines and agreements. They are, however, an excluded component of WTO agreements or rulings [24]. This is despite efforts of the ILO to work with the WTO and IMF on technical reports to highlight links between social and trade policy that influence decent work. Furthermore, countries first need to establish their own legally binding social policy to enshrine these agreed-upon conventions into legislative commitments, and second, countries are only required to self-report on their implementation to the ILO. This leads to inconsistent and incomplete reporting by countries, and limited knowledge is available about the extent to which countries are adhering to policies that promote decent working conditions on the ground [11].

Kingdon argued that only once there is convergence across the problems, policies and politics streams can a problem or an issue emerge on the policy agenda [14]. We suggest that convergence across these three streams has not quite occurred at the global level. The evidence base for problems is debated, policies are multiple and complex and diverse organizational interests challenge organizations to sustain attention on health and social issues. For example, the complex set of macro-economic, labour, social, and education policies that must be coordinated to promote workers’ well-being points to the political challenge of keeping this issue on the policy agenda in this globally contested space with a “high degree of pluralisation of actors and multiple and contested modes of authority than is usually the case at national levels of policy making” [25].

Achieving greater policy coherence and integration across diverse organizations remains an ongoing challenge in a social and political space shaped by the interactions between organizations with different agendas, values, assumptions, and operating under different rules [25,26]. For example, the three organizations do not operate within the same multilateral system - the ILO and WHO do, whereas the World Bank does not. Global institutions need to stay nimble to take advantage of catalytic events that can mobilize attention towards issues with otherwise limited agenda access. These events can also provide opportunities to reframe the discourse about decent work where there is greater balance between socially-oriented objectives and market needs.

As Baumgartner and Jones observed, agenda setting can occur during periods of significant change brought on by catalytic events, which mobilize interests [16]. Events such as the economic crisis helped to reignite concern and garner greater political attention toward decent work. They underscore that the pursuit of decent work does not have to be at odds with economic growth and development. Events create disruptions in the policy equilibrium and make agenda setting more discernible from the overall policy development process, in contrast to periods of stability when it is difficult to separate out agenda setting from the policy-making process. Events lead to the identification of windows of opportunity when agenda setting processes can best be studied. It remains to be seen whether the agenda setting effects of disruptions in the policy equilibrium, such as the recent economic crisis, can be sustained to further benefit workers’ health and well-being. The dominant normalized discourse that markets always know best continues to challenge the legitimacy and policy effects of many organizations in the global policy arena. Such a perspective privileges market-based solutions where nothing in theory is free from commodification - workers are commodities (i.e. goods and services to be purchased) and the policies enacted in their name act as instruments of commodification [27].

### Limitations

The small number of participating organizations was a limitation of the study in that the results do not include potentially more diverse organizational perspectives that could also influence agenda setting related to decent work. These include other UN organizations and groups such as the international labour rights forum who support workers’ rights, but also non-governmental organizations and unions all advocating for decent working conditions or organizations and coalitions who may oppose these objectives. Further research is needed to elucidate other organizational perspectives and their influence on global agenda setting regarding decent work. The selection of key informants also presented some theoretical and methodological challenges. Informants can speak on their own accord as well as for the organizations they represent [28]. In this study, it was not always readily apparent from whose perspective KIs were speaking, despite attempts to probe for distinctions. That said, informants did shed light on how the use of different language (e.g. decent work vs. good jobs) influences or not the policy agendas of their respective institutions. They were generally quite willing to talk about individual and organizational viewpoints on decent work and the DW Agenda.

The second limitation related to the timing of interviews, which were conducted when at least one participating organization was going through restructuring, while others were facing imminent changes in leadership. In addition, interviews were conducted before the October 2012 public release of the World Bank’s 2013 World Development Report on Jobs [23]. These temporal factors influenced in some instances our ability to recruit participants and, to some extent, KIs’ ability to represent the most recent position of their organization.

## Conclusion

In this article, we present the findings from 16 interviews with representatives from the ILO, WHO and World Bank regarding decent work and the Decent Work Agenda. Our evidence furthers understanding of how an issue like decent work can influence the policy agendas of global institutions, using the Decent Work Agenda as an illustrative example. In a nutshell, the findings tell us that agenda setting is related to: the saliency of decent work as an issue that is garnering attention and mobilizing interests especially after the economic crisis; increasing congruence between the Agenda and the organizational objectives of others; and, policy coherence. Despite the ILO’s strong and steady leadership and commitment to advance this Agenda globally, we conclude that the DWA has not been wholly incorporated by the WHO and the World Bank although there is evidence of policy coherence at least within the UN context. Since 1999, agenda setting related to decent work has been realized incrementally and expressed through policy coherence enabled through formal global governance mechanisms (e.g. UN coherence system process), and through influence on the discourses and practices of individual organizations in particular the WHO. Organizational precedents, joint collaborative structures and endorsements seem to positively support agenda setting. Catalytic events such as the recent economic crisis and organizations’ strategic response to them can lead to a potential paradigm shift away from the markets *always* knowing what is best towards social protection, sustainable development and fair globalization. Conversely, these catalytic events provide an impetus for how actors frame and reframe their messages about decent work (in this case) in relation to the global economic downturn. The global policy arena remains fluid and dynamic and is shaped by the emergence of new partnerships and initiatives, changes and catalytic events that affect and engage a plurality of actors. Efforts such as the Social Protection Floor Initiative [21] and the more recent UN Platform for Monitoring the Social Determinants of Health [29] or the arrival of a new leader at the ILO could each give new legs to the Decent Work Agenda. It remains to be seen whether the DWA will maintain its currency on the global policy agenda, take on different discursive forms in response to the changing sociopolitical context, and meaningfully contribute to improvements in working conditions globally.

### Endnotes

^a^A description of the Decent Work Agenda can be found on the ILO website at: http://www.ilo.org/global/about-the-ilo/decent-work-agenda/lang--en/index.htm.

^b^These interviews were conducted before the public release of the World Bank’s 2013 World Development Report on Jobs in October 2012.

^c^http://www.undg.org/content/about_the_undg/undg_members. Note that the World Bank has observer status while the ILO and WHO are members.

## Abbreviations

ILO: International Labour Organization; WHO: World Health Organization; WB: World Bank; DWA: Decent Work Agenda; DW: Decent work; KI: Key informant; KIs: Key informants; UN: United Nations.

## Competing interests

All authors declare that they have no competing interests.

## Authors’ contributions

EDR conceived the study, conducted all key informant interviews and led the interpretation and analysis of data, and wrote first drafts of the manuscript. JEC and DCC contributed to the study design and the interpretation of results, along with critical revisions of drafts of the paper. All authors read and approved the final manuscript.
